# Brain folding shapes the branching pattern of the middle cerebral artery

**DOI:** 10.1371/journal.pone.0245167

**Published:** 2021-01-07

**Authors:** Diego E. Shalom, Marcos A. Trevisan, Arka Mallela, Maximiliano Nuñez, Ezequiel Goldschmidt

**Affiliations:** 1 Physics Institute of Buenos Aires (IFIBA) CONICET, Buenos Aires, Argentina; 2 Department of Physics, University of Buenos Aires (UBA), Buenos Aires, Argentina; 3 Department of Neurosurgery, University of Pittsburgh Medical Center, Pittsburgh, PA, United States of America; 4 Department of Neurosurgery, El Cruce Hospital, Provincia de Buenos Aires, Argentina; 5 Division of Molecular Neurobiology, Department of Medical Biochemistry and Biophysics, Karolinska Institute, Stockholm, Sweden; Columbia University Medical Center, UNITED STATES

## Abstract

The folds of the brain offer a particular challenge for the subarachnoid vascular grid. The primitive blood vessels that occupy this space, when the brain is flat, have to adapt to an everchanging geometry while constructing an efficient network. Surprisingly, the result is a non-redundant arterial system easily challenged by acute occlusions. Here, we generalize the optimal network building principles of a flat surface growing into a folded configuration and generate an ideal middle cerebral artery (MCA) configuration that can be directly compared with the normal brain anatomy. We then describe how the Sylvian fissure (the fold in which the MCA is buried) is formed during development and use our findings to account for the differences between the ideal and the actual shaping pattern of the MCA. Our results reveal that folding dynamics condition the development of arterial anastomosis yielding a network without loops and poor response to acute occlusions.

## Introduction

Theoretical models for optimal transport networks have been extensively investigated. These models solve, for a network built using a fixed amount of resources (pipes), the most efficient transport system allowing for oscillations in flow [1, 2]. For example, the material cost of a network can be reduced using narrow vessels, but this occurs at the expense of a high consumption of energy to maintain the flow of a viscous fluid such as blood. Wider vessels, on the contrary, dissipate less energy but require a greater amount of material to be built. Optimal networks arise from a balance between energy consumption and material resources [3]. Theoretical models for optimal transport networks can compute these optimal networks [1, 4]. These models provide instances of locally optimal networks that show robust topological properties, notably tree-like structures and loops that balance the benefit of creating bridges, capable of rerouting flow in response to environmental challenges, against the cost of creating those new channels [1, 5, 6].

Although this problem has been solved for simple geometries, such models fall short of showing how an optimal vascular network would fit an irregular surface with peaks and valleys, a scenario relevant to explain the blood supply to the brain. Brain folding and the development of the subarachnoid vascular network that irrigates it are concurrent and interdependent phenomena. In contrast to leaf venation, the main arteries feeding the brain surround the substrate they irrigate. Changes in the outer surface of the brain condition the geometry of the blood vessels; thus, the vascular network folds with the brain. How the folding process impacts the development of the brain arteries is still unknown.

Here, we generalized optimal transport to any folded contour and applied it to the geometry of the major depression in the human brain, the Sylvian fissure, irrigated by the middle cerebral artery (MCA). The MCA was chosen for study rather than the anterior cerebral artery (ACA) or posterior cerebral artery (PCA) because its trajectory would potentially allow for anastomosis and loop formation at a minimum cost. Both the ACA and PCA have a more typical terminal branch configuration, without double 90 degree turns and backtracking, making loop formation inefficient. Although there is redundancy within the proximal brain circulation, provided by the circle of Willis; we studied the MCA because, lying distal to it, any acute obstruction in this vessel develops into a brain infarct in almost every case.

We computed simple observables to compare the configurations of these optimal transport networks with normal human anatomy. By using a collection of fetal MR scans, we described how the Sylvian fissure folds into its adult form and explain why, against all odds, the vascular network supplying the brain falls far away from optimal configurations. This, in turn, explains the unexpected abundancy of non-anastomotic (terminal) configuration of the arteries feeding this part of the brain, exposing major real estate to shortage of blood supply and strokes [7].

## Results

### Optimal transport in the Sylvian fissure

**Fig 1:**
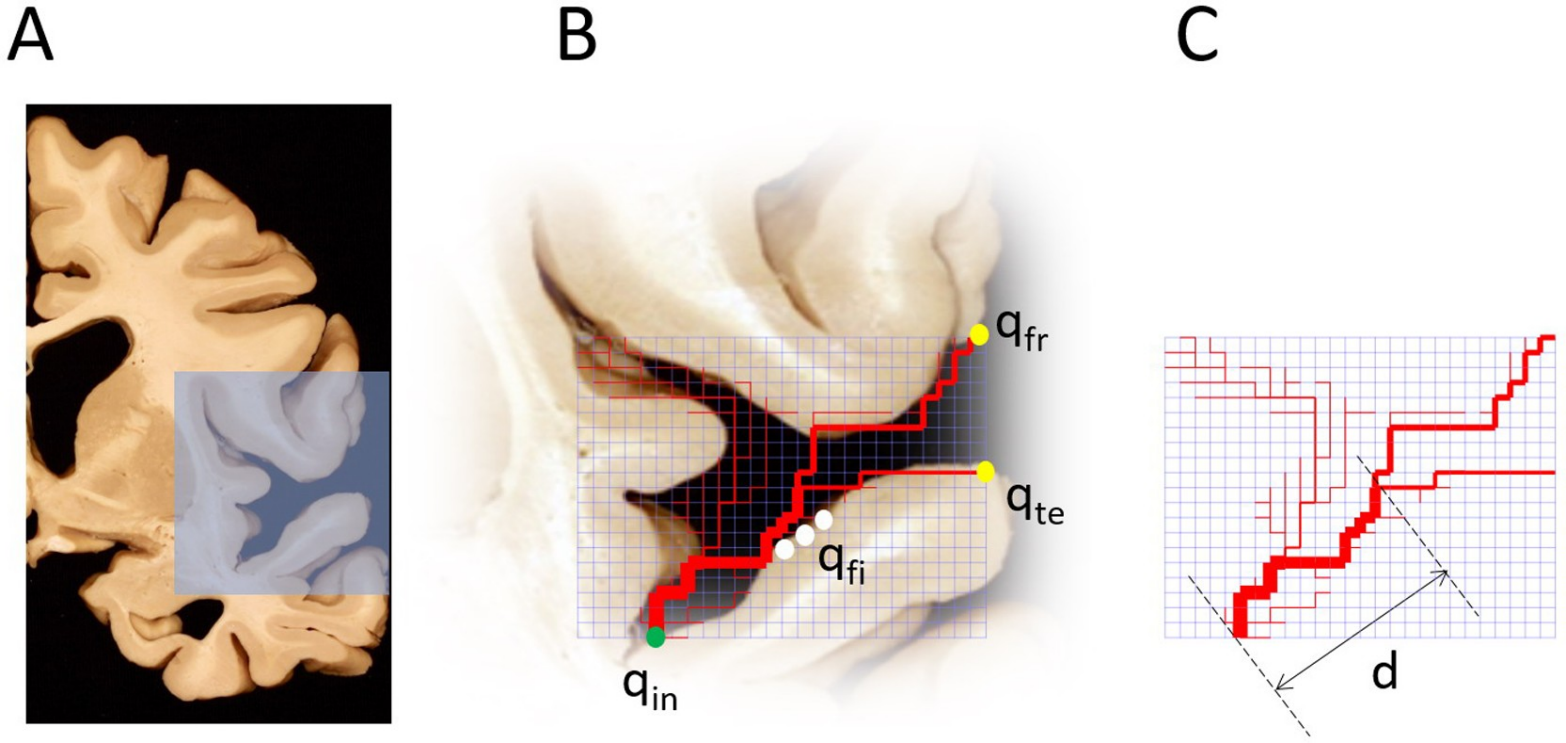
Simulating optimal irrigation in the Sylvian fissure. We modeled the geometry of the lateral sulcus (A) using a plane 80×90 network connected to first neighbors (B). The nodes at the sulcus contour (white) were set as fluctuating sinks of identical mean value *q*_*fi*_ ∼ *N*(1,*σ*); a source *q*_*in*_ was set at the entrance of the sulcus (green) and sinks *q*_*fr*_ and *q*_*te*_ that represent the outflow towards the frontal and temporal lobes (yellow) were set to satisfy the conservation of flow, *q*_*in*_ + *q*_*fr*_ + *q*_*te*_ + ∑*q*_*fi*_ = 0. Two observables were computed to characterize the optimal transport networks (C): the distance *d* at which the MCA bifurcates and the number of loops.

We investigated the topological properties of optimal networks feeding the Sylvian fissure (Fig 1B). In this model, uncorrelated fluctuating sinks were located at the nodes of a grid along the sulcus profile, identified by the white dots in Fig 1B. We located a single source for the MCA at the origin of the fissure (green dot) and two sinks (yellow dots) were fixed at the grid exits, representing the outflow towards the frontal and temporal lobes.

To explore the dependence of optimal transport networks on these vascular variables, we computed two simple magnitudes: the distance at which the MCA branches within the Sylvian fissure, and the number of loops in the network (Fig 1C). The first is a simple observable that can be immediately related to the brain anatomy, and the second, a measure of resiliency to blockage of the vascular system [8]. We defined the following two variables to explore the dependence of optimal networks on vascular parameters: *r*_*out*_ = (*q*_*fr*_ + *q*_*te*_)/*q*_*in*_ represents the fraction of the flow that leaves the fissure with respect to the flow that feeds the fissure, and *r*_*fr*_ = *q*_*fr*_/(*q*_*fr*_ + *q*_*te*_) represents the amount of flow irrigating the frontal lobe with respect to the available outflow.

**Fig 2:**
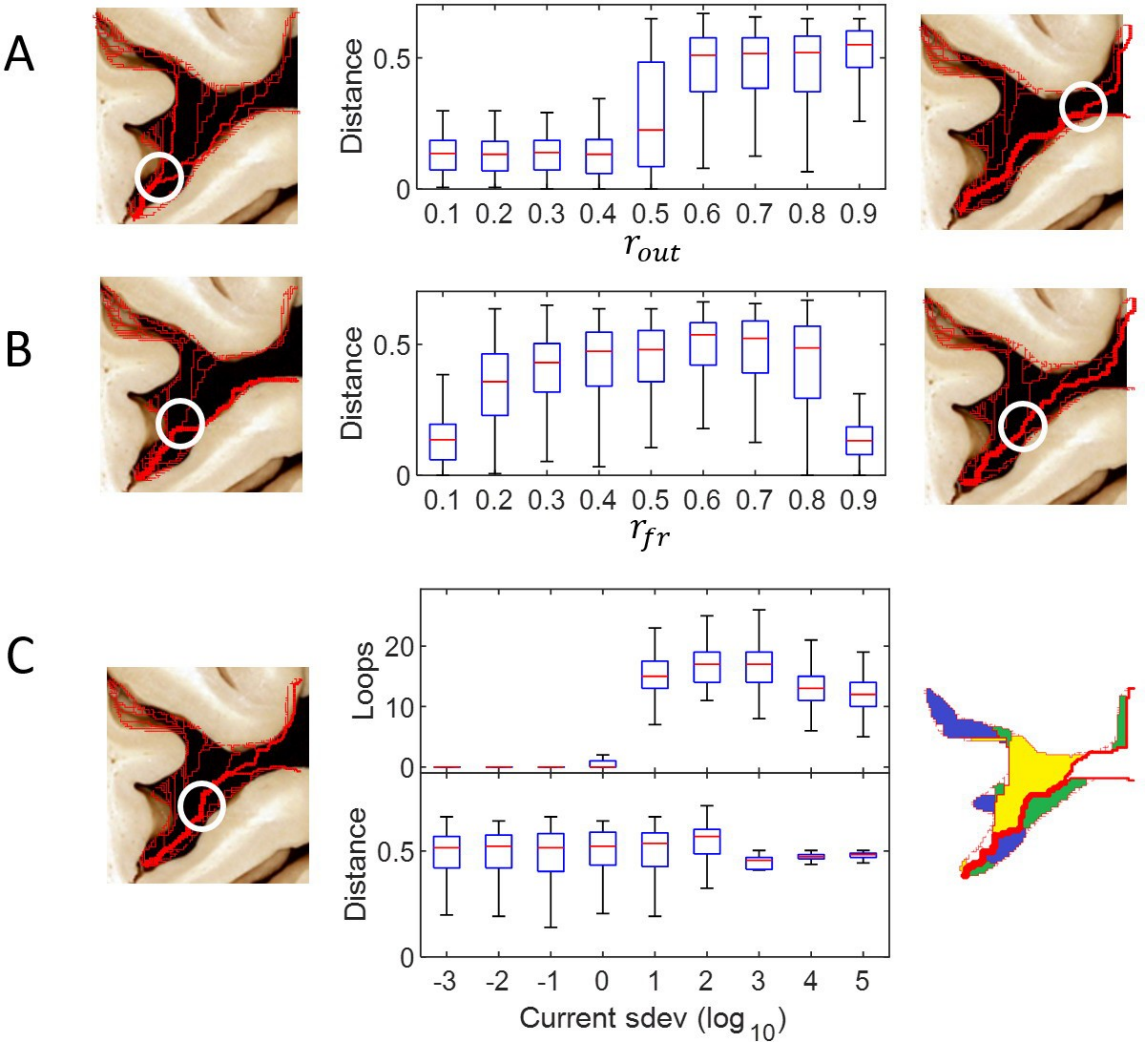
Dependence of network structure on vascular conditions. We explored the effects of three variables in the structure of the network: (A) the fraction *r*_*out*_ of the blood that leaves the Sylvian fissure, (B) the fraction *r*_*fr*_ of the blood that leaves the fissure to irrigate the frontal lobe and (C) the current variability (standard deviation *σ* from unity currents feeding the fissure). For each ratio, we generated 200 locally optimal networks, for which we computed the bifurcation distance (the exit of the fissure is at distance 1) and the number of loops in the vascular network. Colors in the right bottom panel indicate independent loops.

Fig 2 summarizes our results. Panel A shows that when the fraction *r*_*out*_ of blood flow leaving the fissure, is increased over 0.5 (where half of the MCA flow is consumed within the fissure), a steep increase in the bifurcation distance is attained, to roughly half the fissure depth (t-test of *r*_*out*_ < 0.5 vs *r*_*out*_ > 0.5: p<0.0001, t(1598) = −62.54). Instead, when the fraction of the outflow towards the frontal lobe *r*_*fr*_ is increased, the bifurcation distance shows an inverted U shape, with a large plateau around half of the fissure depth, as shown in panel B (t-test of $r_{fr}<0.25\;vs\;r_{fr}\in \left[0.25,0.75\right]:p\;<\;.0001,\;t\left(1398\right)\;=\;-22.83;\;r_{fr}\in \left[0.25,0.75\right]\;vs\;r_{fr}>0.75\;:p\;<\;.0001,\;t\left(1398\right)\;=\;18.04$). While the number of loops is not affected by *r*_*out*_ and *r*_*fr*_, fluctuations in the blood demand above a threshold value of *σ* ∼ 1 rapidly increase the number of loops, as shown in upper panel C (t-test of *σ* < 1 *vs σ* > 1: *t*(1598) = 18.5, *p* < 0.0001). This increase was expected [2], as the network creates different redundant paths to account for high variations in the blood supply within the fissure. Finally, fluctuations do not affect the bifurcation distance, as shown in lower panel C (t-test of *σ* < 1 *vs σ* > 1: *t*(1598) = −0.79, *p* = 0.43).

A single variable was explored at a time, and the others were kept constant at the following values: *r*_*out*_ = 0.7, outflow to *r*_*fr*_ = 0.6 and *σ* = 10^−3^. The flow proportion constants were set by estimating flow distribution to a certain area as a proportion of the cortical surface. The value of *σ* = 10^−3^ was chosen to represent a negligible variation in flow.

In addition to these findings and regardless of *r*_*fr*_ and *r*_*out*_, the computed configuration exhibits an early ascending branch that irrigates the space between the frontal lobe and the insula (the frontal aspect of the insular cleft (Fig 2).

Taken together, these analyses show that optimal irrigation requires that MCA bifurcates well inside the sulcus and the presence of an early ascending branch. None of these features are present in normal anatomy, which falls far away from our calculated optimal configuration. This apparent inconsistency has a simple explanation. The Sylvian fissure forms by the convergence of two distant poles of the human embryonic brain and the three-dimensional proximity of its elements only exists very late in gestation or even after birth, when the vascular network is incapable of major reconstruction.

### Normal adult human anatomy

In order to characterize the anatomy of the insula and the middle cerebral artery, an anatomical cadaveric dissection of 10 injected human heads was performed.

**Fig 3:**
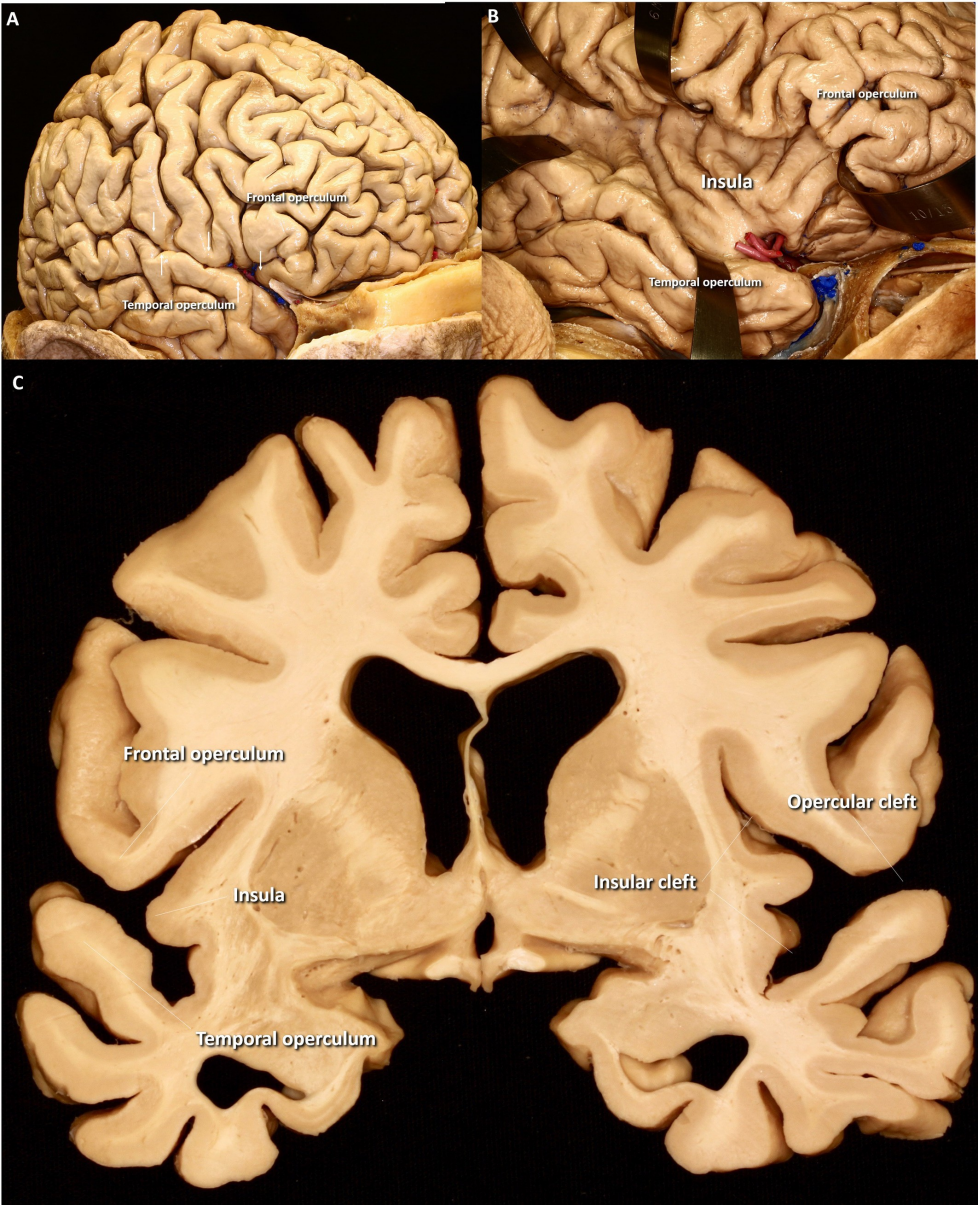
Anatomy of the insula and its limiting opercula. The Sylvan fissure (arrows) is the most distinctive fold of the lateral hemispheric surface (A). Although it resembles other cortical sulci the Sylvian fissure runs deeper than any other and connects the lateral hemispheres with the basal cisterns. The frontal and temporal lobe regions limiting this fold are named opercula and bound the superior and inferior aspect of the fissure; its medial aspect is formed by the insula, a unique cortical region with distinctive convergent shallow folds (B). The insula and the opercula limit the divisions of the fissure into its principal components (C).

The Sylvian fissure is the subarachnoid space lying between the frontal and temporal lobe. The insular cortex forms the deeper aspect of it, while its surface forms the most characteristic depression of the lateral hemispheric surface (Fig 3). The space immediately deep to the surface is a division of the Sylvian fissure called operculoinsular compartment [9]. This space is further divided into an opercular cleft (between the frontal and temporal lobes) and an insular cleft with two rami between the insula and the medial part of the frontal and temporal lobes respectively (Fig 3). The behavior of the middle cerebral artery in this space is the focus of this paper [10–12].

The MCA is contained within the Sylvian fissure. As the largest telencephalic artery, it irrigates the insula, the opercula and most of the lateral hemispheric surface. The geometry and branching pattern of the MCA have been extensively described [13, 14]. The M1 segment of the MCA runs laterally from the carotid bifurcation to reach the Sylvian fissure, where it turns 90 degrees and immediately bifurcates into a superior and an inferior trunks. A minority of people exhibit a trifurcation with an additional middle trunk [9, 15].

**Fig 4:**
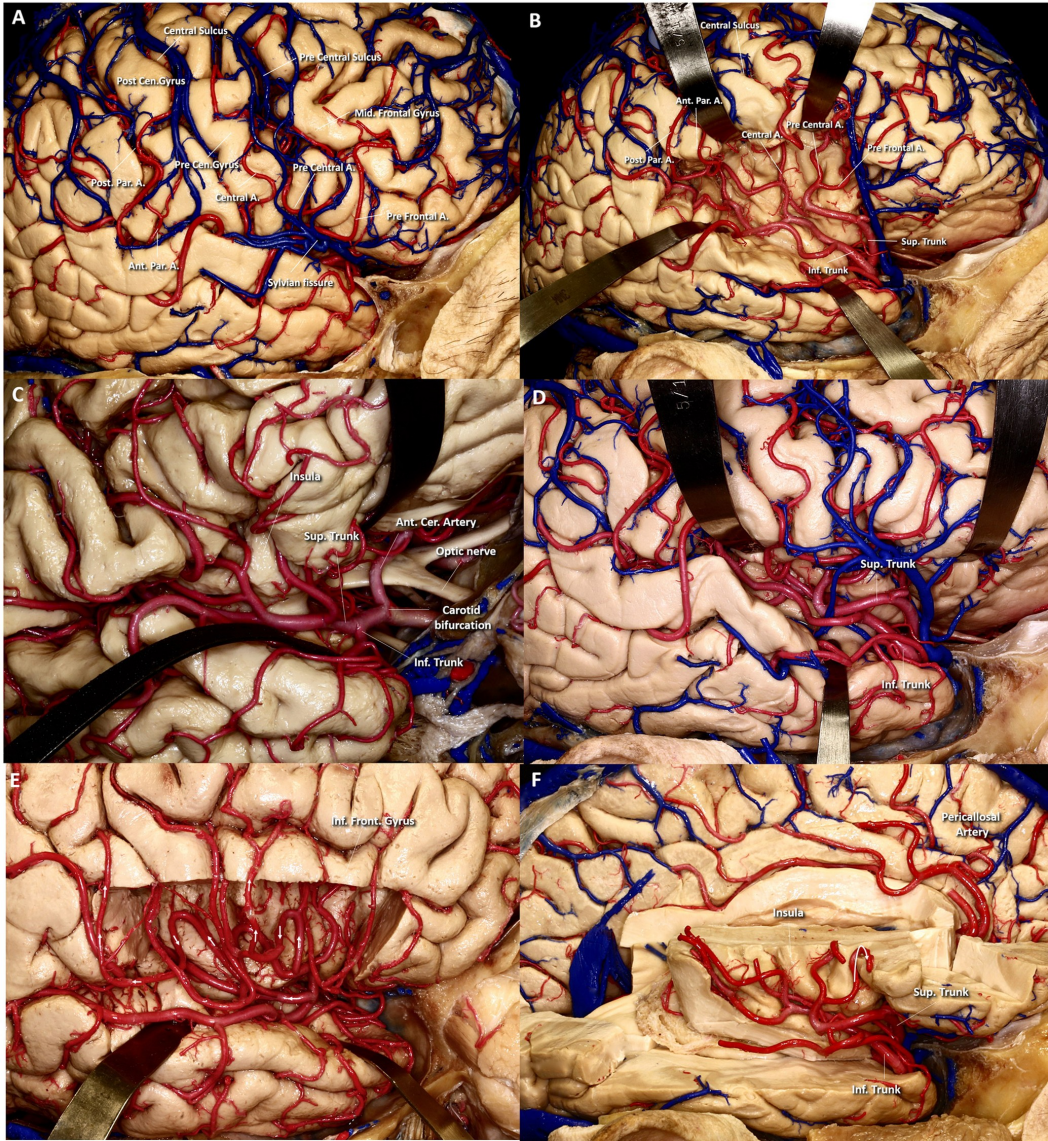
Anatomy of the Middle Cerebral Artery (MCA). The cortical branches of the middle cerebral arteries are depicted on this lateral view of a right hemisphere (A). As shown here, the MCA irrigates most of the lateral surface of the frontal and parietal lobe and the entire lateral temporal surface. Despite their proximity in the Euclidean space, no anastomotic vessels exist between the frontoparietal and temporal vessels and no arteries run across the Sylvian fissure. The distribution of the MCA vessels within the fissure are shown in (B). The lower trunk branches follow the superior aspect of the temporal lobe to reach the lateral subarachnoid space; the superior trunk climbs up the insular surface to make a complete turn at the top of the insular cleft. (C) and (D) show the apparent plexiform nature of the MCA branches that, on close examination, exhibit no anastomotic vessels or connections between the superior and the inferior trunk systems. (E) shows a lateral view of a right hemisphere in which the inferior frontal gyrus has been removed allowing a clear vision of the 180 degree turn of the MCA at the top of the insular cleft (arrows), the insular surface can be seen in the depth. On (F) the entire right hemisphere except for the central core and the inferior aspect of the temporal lobe has been removed. The 180˚ turn of the superior division of the MCA at the top of the insular cleft is again visible. The branches of the anterior cerebral artery are visible at the medial aspect of the left hemisphere.

The inferior trunk follows the contour of the fissure close to the temporal lobe to finally dive into the lateral surface of the temporal lobe (Fig 4). The superior trunk climbs up, following the insular cortex until reaching the roof of the insular cleft, where it turns 90 degrees twice to head back down in search for the opercular cleft where another 90-degree turn puts it closer to the lateral frontal surface that it will supply. Despite the many variations described and the proximity of the MCA branches, they never anastomose or cross the Sylvian fissure [16]. The insular cleft has no dedicated arteries, thus elongating the MCA up and down, increasing its length but adding no anastomotic escape pathways if its patency is compromised. A reason for the discrepancy between our optimal network and the normal anatomy can be derived from the embryological process that ultimately yields the fissure.

### Convergence of distant brain poles form the Sylvian fissure

**Fig 5:**
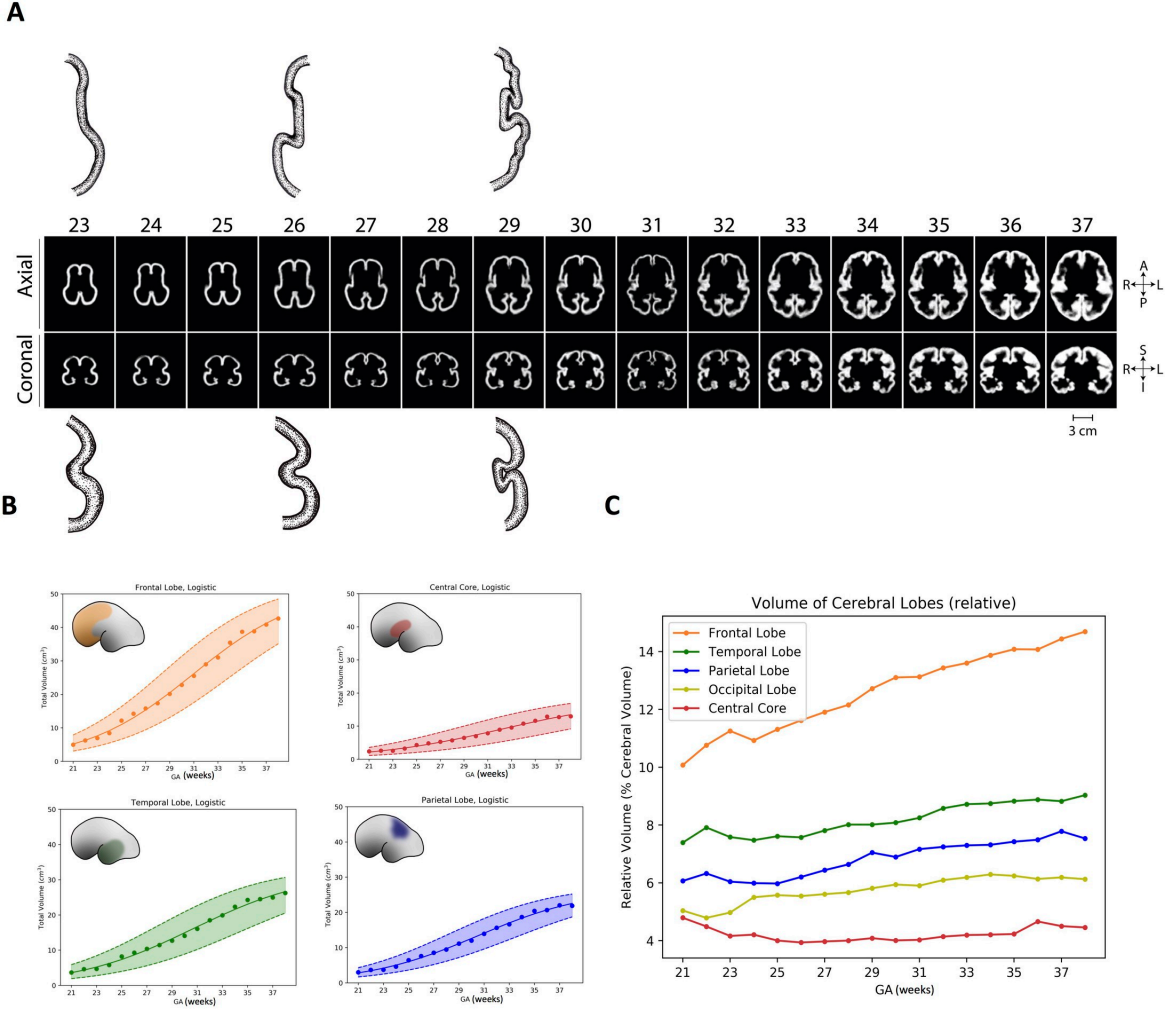
Development of the Sylvian fissure. (A) represents an overview of the growth of the human brain *in utero* from post conceptional week 23 to 37. (B) represents the volumetric growth of the frontal lobe, central core, temporal and parietal lobes respectively. Logistic denotes the type of fit that was used to calculate the error in the figure (C) shows volumetric growth relative to total intracerebral volume at a given point. The insula and the central core grow at a slower rate than any other region and represent the smallest relative volume throughout gestation (ANCOVA controlling for side and gestational age; F(4, 173) = 123.61, p < 0.0001).

A normalized map of brain development, derived from 81 normal fetuses during peak folding, shows that the Sylvian fissure evolves from a flat surface (the central core and the insula) delimited by an anterior and posterior protuberances in the telencephalic bubble, which will become the frontal and temporal lobes, respectively. The poles grow over the insula from a distant position to close the subarachnoid space in between, forming the fissure. Thus, the proximity of frontal, temporal, and insular lobes in the Euclidean space is such only very late during gestation (Fig 5).

**Fig 6:**
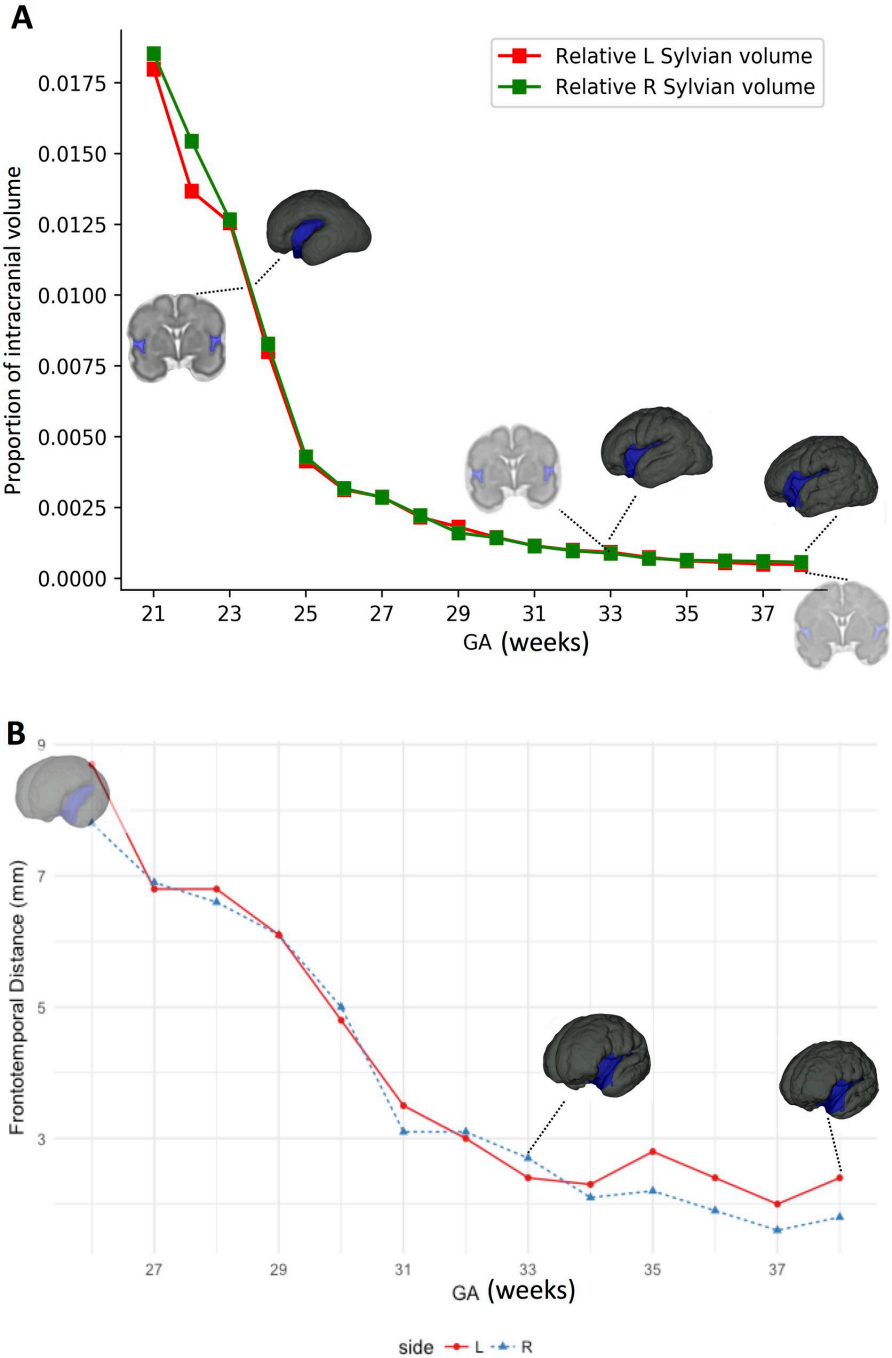
The relative volume of the Sylvian fissure contracts over time. (A) shows that the volume of the fissure (expressed as a proportion of the total intracranial volume) diminishes over time. A three-dimensional reconstruction and a coronal cut of the averaged MR images from the Gholipour atlas at weeks 24, 33 and 38 are depicted. The fissure volume is painted in light blue. (B) represents the absolute distance between the frontal and temporal lobes over time. The growth of these two lobes over the insula produces a graph in which the fronto-temporal distance diminishes over time, consistent with the relative volume contraction. These two lobes grow into each other and come in close contact only at the very end of gestation.

To formalize the abovementioned observations, we analyzed the total and relative volume (with respect to the cerebrum) of the central core and compared it the parenchyma that would ultimately form the Sylvian fissure; including the frontal, temporal, and parietal lobes (Fig 6). The volume of the frontal lobe was 5.0 cc at 21 weeks (10.0% of cerebral volume) enlarging 8.5-fold to 42.7 cc (14.7%) at the end of gestation. Likewise, the temporal and parietal lobe enlarged by 7 and 7.5 times with no significant differences between left and right hemispheres. In contrast, the insula and central core developed from 2.4 cc (4.8% of cerebral volume) at 21 weeks to only 12.9 cc (4.5%) at Week 38, a 5.5-fold increase. Both the magnitude and rate of volumetric growth were significantly higher in the lobes compared to the central core (ANCOVA controlling for side and gestational age; F(4, 173) = 123.61, p < 0.0001). These findings show that that the limits of the fissure overgrow the central core, consistent with the hypothesis that the frontoparietal and temporal lobes converge over the insula and progressively close the Sylvian fissure.

## Discussion

The Middle Cerebral Artery is first identified when the embryo is 4 weeks old and 12 mm long. It appears as a plexiform network that branches from the Internal Carotid Artery covering the lateral hemispheric surface and is shaped similarly to a spider web with multiple intra-arterial anastomosis [7, 17]. This redundant configuration dramatically changes as the brain grows and folds, to ultimately yield a vessel in which terminal circulation is the norm and escape routes are lacking. This paper provides an explanation for this odd phenomenon.

To do so, we calculated a network that irrigates a surface at a minimum expense. Although a system of narrow vessels is less expensive, the viscosity of the blood produces a high consumption of energy to maintain the flow. Wider vessels, on the contrary, dissipate less energy but require a greater amount of material. Optimal networks arise from the interplay between energy and material consumption, from which specific network architectures arise. This has been explored in the case of the human cardiovascular system, for which material-cost optimizations are the strongest predictor of vascular branching in the human head and torso [18].

Here, we simulate the irrigation of a complex brain surface under the constraints of optimal transport. Theoretical models naturally provide us with ensembles of locally optimal networks compatible with a given set of vascular conditions. We therefore constructed statistically significant observables that can be readily compared with the normal brain anatomy. Our simulations show that, for a wide range of vascular regimes, including normal brain conditions, the MCA bifurcates well inside the fissure, with an early ascending branch that irrigates the space between the frontal lobe and the insula; all features that differ significantly from the normal anatomy.

Brain folding occurs in different dimensions. The development of the sulci and gyri within a lobe is the result from biological and physical phenomena, ultimately bending the brain surface by tangential expansion; the folding of the frontal and temporal lobe over the insula, to form the Sylvian fissure, is a completely different event [19]. Bush et al. previously suggested that the Sylvian fissure appears by convergence of the anterior and posterior poles of the embryonic telencephalon and proposed a convergence index for the adult brain, calculated as the Euclidean distance/transcortical distance ratio, that returns its highest values around the fissure [20]. Recently, Mallela et al. showed that, contrary to intralobar sulci, the subarachnoid space in the Sylvian fissure contracts as the fissure grows; the edges of this singular fold do not bend, they converge [21]. Here, we show that the distance between the frontal and temporal lobes diminishes with gestational time as does the volume of the Sylvian fissure as a result of the overgrowth of the opercular lobes over the insula and the central core. The surfaces of the frontal, insular and temporal lobes only meet each other late during gestation, when the building blocks of the vascular network lack anastomotic capacity, thus explaining why they develop as independent, and not interconnected systems. Like two cars colliding, the frontal and temporal lobes have little to do with each other until it is too late to majorly modify their respective vascular networks into one.

Our findings do not come without limitations. The assumption that optimal networks building principles apply to brain embryology has not been proved. While evidence for other systems exists, a similar analysis for the central nervous system is lacking, and obtaining such proof may not be feasible given the difficulty in imaging developing brain vessels and the logical lack of tissue for direct examination. Similarly, flow changes and other major circulation variables would be close to impossible to attain during development.

It also noteworthy that both carotid arteries communicate with the posterior circulation by the circle of Willis, an anastomotic network that can redirect flow and prevent strokes after carotid and vertebral artery occlusions, and global hypoperfusion. An acute obstruction distal to it, will, on the other hand, yield a brain infarct in almost every case.

Our results, nevertheless, provide new insights into how the folding of the brain shapes its irrigation, explaining the lack of redundant pipes in a critical region and exposing the brain to strokes after the acute occlusion of any segment of the middle cerebral artery.

## Methods

### Optimal transport network within the Sylvian fissure

To simulate the irrigation of the Sylvian fissure we set a plane square network with nodes connected to first neighbors (Fig 1B). We placed fluctuating sinks of unity mean value *q*_*fi*_ = *N*(1,*σ*) along the fissure surface (white dots). These currents model the fluctuating demand of blood in the brain surface, and are a main source of loops [2, 8]. An inward flow *q*_*in*_ > −∑*q*_*fi*_ represents the blood injected into the sulcus by the middle cerebral artery (green dot); two sinks *q*_*fr*_ and *q*_*te*_ were fixed at the exit of the grid (yellow dots), representing the outward flows towards the front and temporal lobes respectively. These quantities satisfy *q*_*in*_ + *q*_*fr*_ + *q*_*te*_ + ∑*q*_*fi*_ = 0. Nodes outside the subarachnoid space were disconnected from the network.

For a given set of MCA inflow *q*_*in*_, outward flows *q*_*fr*_ and *q*_*te*_, and fluctuations *σ*, we obtained the conductances for a (locally) optimal transport network following the procedure described in [2]. The initial random conductances *k*_*kl*_ between first neighbour nodes *k* and *l* in the network were updated according to the rule

$$k_{kl}=K\frac{{\langle q_{kl}^{2}\rangle }^{\frac{2}{3}}}{{\left(\sum_{m,n}{\langle q_{mn}^{2}\rangle }^{\frac{1}{3}}\right)}^{2}}$$

until iterations produced no further changes, as described in [2]. The resulting optimal network is visualized by assigning an edge width proportional to the square root of the conductance *k*_*kl*_ between nodes *k* and *l* of the network (Fig 1C). In order to study the dependence of the results with the spatial resolution, simulations were performed on networks of 27×21, 40×34, 68×60 and 80×90. Increasing the size of the grid led asymptotically to the statistics shown in Fig 2, obtained for 68×60 and higher. We used networks of 80×90 throughout this work, which ensured an adequate spatial resolution, since further increase of grid size produced statistically equivalent results. Optimal transport simulations were calculated using Matlab.

### Vascular parameters and network observables

We defined the following two variables to explore the dependence of optimal networks on vascular parameters: *r*_*out*_ = (*q*_*fr*_ + *q*_*te*_)/*q*_*in*_ represents the fraction of the flow that leaves the fissure with respect to the flow that feeds the fissure, and *r*_*fr*_ = *q*_*fr*_/(*q*_*fr*_ + *q*_*te*_) represents the amount of flow irrigating the frontal lobe with respect to the available outflow.

Given a set of vascular variables *r*_*out*_, *r*_*fr*_ and *σ*, a family of 200 locally optimal networks was generated, from which two simple observables were computed: the number of loops, which is a measure of the robustness of the network under damage, and the distance at which the MCA bifurcates inside the fissure (Fig 2).

### Anatomical dissections

Although the normal human anatomy of the middle cerebral artery and its variants has been extensively investigated, we produced dissection pictures for illustration purposes. Anatomic cadaveric specimens were obtained from Science Care (AZ, USA) from routine autopsy procedures. The heads were fixed with paraformaldehyde; the veins and arteries were injected with blue and red colored silicone (Dow Corning Corp.) respectively, Thinner 200 (Dow Corning Corp.), and RTV catalyst (Dow Corning Corp.). Dissection was carried out in a manner which exposed and preserved the lateral hemispheric surface, insula, Sylvian fissure and the subarachnoid vascular network. Photographs were taken for documentation. The study was approved and supervised by the University of Pittsburgh office of oversight of anatomical specimens, which consists of a committee of experts that reviewed and approved this study (CORID #764).

### Description of the developmental dynamics of the Sylvian fissure

#### Fetal brain MRI atlas, registration and validation

An atlas of fetal brain MRIs was used. The atlas includes imaging from 81 healthy fetuses and was developed by Gholipour et al., it spans gestational weeks 21 to 38. [22]. The acquisition details and the full content of the atlas can be accessed at http://crl.med.harvard.edu/research/fetal_brain_atlas/. The Symmetric Normalization (SyN) algorithm in the Advanced Normalization Tools (*ANTs*) package, that registers weekly image pairs (Week 24 to 25, etc.) was used to quantify week-to-week changes as described by Mallela et al. [21] The original segmentations in the Gholipour atlas [22] was used as the ground truth to validate the accuracy of our registration.

In addition to the originally described structures by Gholipour et al. we algorithmically generated segmentations for the Sylvian fissure, defined as the CSF space lying between the margins of the frontal operculum, temporal operculum, and supramarginal gyrus, by using the procedure described by Glaister et al. [23], which allows for the identification of cerebrospinal fluid (CSF) voxels lying between predefined brain segments.

The intracranial volume was defined as all intradural volume, the ventricles and brain lobes were defined using the preexisting segmentation [22] and the Sylvian fissure was defined as described before. Volumes were calculated for the Sylvian fissure and limiting brain parenchyma. The relative distance between the frontal and temporal opercula was measured throughout gestation. We utilized ANCOVA (analysis of covariance) to determine whether total and relative volumes were significantly different between the insula/central core and the other lobes, controlling for gestational age and laterality. Imaging examination was performed using the *NumPy* and *NiBabel* packages for Python (Python 3.5). The *pandas* package was used for statistical analysis and data plots were generated using *matplotlib* (Matplotlib version 3.1.1) in Python [24].

#### Visualization

We generated 3D surface images to simplify comprehension of our findings. For figures illustrating sulci and surrounding gyri, we utilized 3D rendering in *itk-snap* [25]. All packages were run on Python 3.7 and utilized under the BSD or GNU license allowing for use in scientific settings.
